# The Effect of Normally Consumed Amounts of Sucrose or High Fructose Corn Syrup on Lipid Profiles, Body Composition and Related Parameters in Overweight/Obese Subjects

**DOI:** 10.3390/nu6031128

**Published:** 2014-03-17

**Authors:** Joshua Lowndes, Stephanie Sinnett, Sabrina Pardo, Von T. Nguyen, Kathleen J. Melanson, Zhiping Yu, Britte E. Lowther, James M. Rippe

**Affiliations:** 1 Rippe Lifestyle Institute, 215 Celebration Place, Suite 300, Celebration, FL 34747, USA; E-Mails: jlowndes@rippelifestyle.com (J.L.); ssinnett@rippelifestyle.com (S.S.); sabrinapardo@hotmail.com (S.P.); vnguyen@rippelifestyle.com (V.T.N.); 2 Energy Balance Laboratory, Department of Nutrition and Food Sciences, University of Rhode Island, Kingston, RI 02881, USA; E-Mail: kmelanson@uri.edu; 3 Department of Nutrition and Dietetics, University of North Florida, 1 UNF Drive, Jacksonville, FL 32256, USA; E-Mail: zhiping.nutrition@gmail.com; 4 Britte Lowther, 4302 39th Street West, Apt # 11, Bradenton, FL 34015 , USA; E-Mail: Britte.lowther@gmail.com; 5 Rippe Lifestyle Institute, 21 North Quinsigamond Avenue, Shrewsbury, MA 01545, USA; 6 Biomedical Sciences, University of Central Florida, 4000 Central Florida Blvd Orlando, FL 32816, USA

**Keywords:** sucrose, high fructose corn syrup, body mass

## Abstract

The American Heart Association (AHA) has advocated that women and men not consume more than 100 and 150 kcal/day, respectively, from added sugars. These levels are currently exceeded by over 90% of the adult population in the United States. Few data exist on longer-term metabolic effects when sucrose and High Fructose Corn Syrup (HFCS), the principal sources of added dietary sugars, are consumed at levels typical of the general population. Sixty five overweight and obese individuals were placed on a eucaloric (weight stable) diet for 10-weeks, which incorporated sucrose- or HFCS-sweetened, low-fat milk at 10% or 20% of calories in a randomized, double-blinded study. All groups responded similarly (interaction *p* > 0.05). There was no change in body weight in any of the groups over the 10-week study, or in systolic or diastolic blood pressure. Likewise, there were no changes in total cholesterol, triglycerides, low-density lipoprotein (LDL), or apolipoprotein B (Apo B). We conclude that (1) when consumed as part of a eucaloric diet fructose—when given with glucose (as normally consumed) does not promote weight gain or an atherogenic lipid profile even when consumed at two to four times the level recently recommended by the AHA. (2) There were no differences between HFCS and sucrose on these parameters.

## 1. Introduction

The prevalence of overweight and obesity have increased significantly over the last 30 years in the United States [1,2,3]. Furthermore, numerous studies have shown that obesity correlates with established risk factors for coronary heart disease (CHD) [4,5]. In this time period consumption of several macronutrients including added fats, flour/cereals and added sugars have increased [6,7,8]. Some recent reports have suggested a potential association between intake of sugars and adverse effects on cardiovascular health [9]. In particular, some evidence in humans suggests that increased intake of sugars may raise blood pressure [10,11,12], although the data are inconsistent [13]. Other studies suggest that increased sugars may raise triglyceride levels and lower high-density lipoprotein (HDL) cholesterol [14,15,16], although once again, the data are inconsistent [17,18]. Studies relating increased consumption of sugar to low-density lipoprotein (LDL) and LDL particle size are also inconsistent [19,20]. Some [21,22], but not all [23,24], studies have also linked higher consumption of added sugar to increased oxidative stress and inflammation. Some epidemiologic studies have suggested a relationship between added sugar consumption and increased risk of obesity [25,26,27]. Other studies have disputed these findings [28,29]. Several recent studies have shown an association between sugar sweetened beverage consumption and increased risk of obesity in children as well as in adults who are genetically predisposed to obesity [30,31,32].

The American Heart Association (AHA) has recently issued a Scientific Statement suggesting that individuals limit added sugars to no more than half of their discretionary caloric allowance; that is, no more than 150 and 100 cal/day for most American men and women, respectively [9]. The AHA statement acknowledges that these recommendations are based on limited data and that relatively few randomized, prospective trials have been conducted. Many of the studies suggesting links between added sugars and cardiovascular disease are either epidemiologic, which establish associations rather than cause and effect [27,28], or experiments comparing pure fructose versus pure glucose at exaggerated levels, a model system that does not reflect the way that sugars are consumed in the normal human diet [33,34,35]. Very few studies have explored the effects of sucrose or High Fructose Corn Syrup (HFCS), the major added sugars in the U.S., at normal population consumption levels [36].

It has been argued that the fructose moiety of both sucrose and HFCS may be particularly worrisome due to its effects on body weight and cardiovascular risk factors [37,38,39]. Both sucrose and HFCS are comprised of roughly 50% glucose and 50% fructose. Sucrose is half fructose/half glucose, while the two major forms of HFCS in common use, HFCS-42 and HFCS-55, are comprised of 42% fructose/53% glucose/5% glucose oligomers and 55% fructose/42% glucose/3% glucose oligomers, respectively [8].

While consumption of HFCS increased significantly in the U.S. between 1970 and 1999 largely at the expense of the sucrose it replaced, its use has been in steady decline for the past decade [40]. It should be noted that fructose and added sugars from all sources have declined in the United States since 1999 [41,42]. At present, the middle 50% of the American adult population for consumption of total fructose represents an intake of 10% of total calories which corresponds to 20% of total calories from added sugars. However, more sucrose is still consumed in the U.S. each year than HFCS, and worldwide, nine times as much sucrose is used than HFCS [8].

It has been postulated that differences in metabolism between glucose and fructose may be a contributing factor to weight gain and obesity, and increased risk of cardiovascular disease and various metabolic conditions such as the metabolic syndrome and diabetes [25,26,27,28]. It has also been postulated that consumption of fructose from any source, whether it be sucrose or HFCS or any of the other glucose/fructose containing nutritive sweeteners, may independently contribute to hypertension, dyslipidemias and insulin resistance [38,39,40,41,42,43].

With this information as background, the current study was undertaken to explore whether two different amounts of sucrose or HFCS consumed at levels equal to the 25th and 50th percentile of the US adult population consumption level for total fructose have any adverse impact on body weight or body composition and blood lipids or blood pressure in a free living, longitudinal study lasting 10-weeks. Overweight and obese individuals were chosen since they are most at risk for these metabolic abnormalities. To our knowledge, this is the first prospective study to examine the effects of added sugars on overweight/obese individuals when sugars are consumed at levels typical of the adult population.

## 2. Experimental Section

### 2.1. Study Design

This study was a 10-week, randomized, prospective, double blinded trial involving overweight/obese subjects between the ages of 25 and 60. The consequences were compared of consuming sucrose or HFCS at 10% or 20% of total calories (25th or 50th percentile population intake levels of fructose) as a component of the usual diet in a free living environment. The study was approved by the Western Institutional Review Board (Project identification code: 20091302, date of approval: 27 July 2009) and the University of Central Florida Institutional Review Board and all participants signed uniformed consent documents.

### 2.2. Subjects

Men and women between the ages of 25 and 60 years of age with Body Mass Index (BMI) 27.0–35.0 kg/m^2^ were recruited. Exclusions included current enrollment in any commercial weight loss program, prescription medicines or supplements for weight loss, or a greater than five pound change in weight during the past three months. Individuals with a history of orthopedic limitations that would interfere with the ability to meet prescribed exercise, a history of heart problems, a history of major surgery within the last three months, clinically diagnosed eating disorders or any gastrointestinal disorder, dietary restrictions or allergies to any component of the diet or which would limit the ability to adhere to dietary requirements of the study were all excluded. Users of tobacco products or individuals consuming more than 14 alcoholic beverages per week were also excluded.

Interested individuals were initially screened over the phone to determine eligibility based on self-reported data. Those who were eligible participated in a clinical screening visit to verify a qualifying height and weight, to rule out undiagnosed hypertension, and to obtain a fasting blood sample for the measurement of glucose, insulin, lipids and C Reactive protein (CRP). Individuals who failed to meet study inclusion criteria were dismissed at that time.

Each subject performed a second screening visit one week later. During this visit, research dietitians assessed participant dietary intake by analyzing a completed three day food record using Nutrient Data System Research (NDS-R) Software (University of Minnesota, Minneapolis, MN, USA). Body composition was determined by Dual X-Ray Absorptiometry (General Electric i-DXA, Madison, WI, USA). All females were required to have a negative serum pregnancy test prior to DXA testing. Repeat measurements were performed after the end of 10 weeks. All cholesterol samples were sent to a certified research-based laboratory with error rates of less than 1%.

### 2.3. Intervention

Throughout the 10-week intervention participants were required to incorporate an amount of low-fat (1% fat) flavored milk sweetened with either HFCS or sucrose (nutritional composition is provided in Table 1), according to their randomly assigned group: 

GROUP #1 (HFCS 10%): 10% of recommended calories provided from HFCS in the milk (equivalent to the 25th percentile for intake of fructose for adults).

GROUP #2 (HFCS 20%): 20% of recommended calories provided from HFCS in the milk (50th percentile for intake of fructose for adults).

GROUP #3 (Suc 10%): 10% of recommended calories from sucrose in the milk (25th percentile intake of fructose for adults).

GROUP #4 (Suc 20%): 20% of recommended calories from sucrose in the milk (50th percentile intake of fructose).

### 2.4. Nutritional Plans

Participants were provided a structured eating plan based on the American Diabetes Association (ADA) exchange lists. Overall caloric level was determined using the Mifflin-St. Jeor calculation for Resting Energy Expenditure (REE) with activity factor included based on responses to a physical activity questionnaire. Daily consumption levels of the test beverage were then calculated according the group assignment (10% or 20% calories from added sugar). Increases in added sugar from the milk were accounted for by reductions in the number of other carbohydrate exchanges allowed. As indicated in Table 1, each carton of milk contained either 11 grams of HFCS or sucrose or 22 g of HFCS or sucrose. The milk was prepared by mixing either HFCS or sucrose solids into a standard 1% milk solution. The amount of solids added were dependent on whether the milk was intended for those at the 10% consumption level (11 g of solids per 240 mL of milk) or the 20% consumption level (22 g of solids per 240 mL of milk). There was no fructose or glucose added to the milk so the only that was present were those in the lactose moiety or the added HFCS or sucrose solids.

Individuals in the 10% HFCS or sucrose groups were given milk containing the lower amount of HFCS or sucrose, while individuals in the 20% HFCS or sucrose groups were given milk containing the higher amount of HFCS or sucrose containing milk. Then, based on the calculated caloric needs of each participant, they were assigned a number of cartons that provided them with either 10% or 20% of their estimated caloric needs from the sweeteners. This could have been as few as two cartons or many as five or six cartons.

**Table 1:** Nutrient composition of milk.

Formulation	1% Fat Milk and Carbohydrate		
**Ingredient**	**1% Low Fat Milk**	**HFCS or Sucrose Solids**	**HFCS or Sucrose Solids**
Consumption Level		10%	20%
Dose	240 mL	11 g	22 g
Fat (g)	2.5	0	0
Carbohydrate (g)	13	11	22
Protein (g)	8	0	0
Calories			
From Milk	110		
From Added Sweetener		44	88
Total Per Serving	110	154	198

HFCS: High Fructose Corn Syrup.

Both participants and investigators were blinded to the type of sugar given to each participant. However, while participants were also blinded to whether they were in a 10% or 20% sugar group, investigators were aware of this information since it was necessary to prescribe the right number of other carbohydrate exchanges. Compliance with diet and test beverage was checked weekly based on completed checklists turned in during a visit in which participants would also have their weight measured and be supplied with another weeks supply of test beverage. Participants were withdrawn if they failed to consume the prescribed milk for five consecutive days or if they failed to maintain an overall level of consumption of at least 80% of that prescribed. All sweeteners were supplied in 1%, low-fat milk (Tetra Pak, Denton, TX, USA) as described above.

### 2.5. Data Analysis

Data were checked for normalcy and analyzed using a two way (time and group assignment) Analysis of Variance with repeated measures. Significant time X group assignment interactions were probed by assessing the within subject change within each of the 4 groups separately. In addition, changes over the course of 10-weeks (week 10-baseline) were calculated and among group differences assessed by one way Analysis of variance (ANOVA). For all analyses the alpha value was set at 0.05. All data were analyzed using SPSS Advanced Statistics V18 (SPSS^®^ Statistics is a comprehensive system for analyzing data, (IBM, Armonk, NY, USA).

## 3. Results

### 3.1. Compliance and Participant Attrition

Of the 86 individuals who started the study, 65 completed the 10-week intervention. Reasons for attrition were varied and included unwillingness to maintain milk consumption levels, inability to attend clinical appointments and investigator determined exclusion due to non-compliance. Only data from the 65 participants who completed the study are presented herein. Baseline characteristics are shown in Table 2. Compliance with prescribed milk intake was very high with over 96% of recommended servings being consumed. Milk consumption among finishers was ranged from two to six eight ounce cups/day in order to achieve either 10% or 20% of calories from HFCS or sucrose.

### 3.2. Dietary Intake

Despite the addition of the sugar-sweetened milk, participants followed a structured dietary plan designed to provide a weight-maintenance level of calories. However, as shown in Table 3, according to analysis of 3 day food diaries caloric intake increased (*p* < 0.001), irrespective of group assignment (interaction *p* = 0.925). There were also increases in carbohydrate and protein and total sugar intake (all *p* < 0.001). Of these, only sugar intake was affected by group assignment (interaction < 0.01). While there was a trend towards a larger increase in consumption for both total and added sugar in the 20% groups compared to the 10% groups statistical significance was only reached for the comparisons between HFCS 20% and sucrose 10% for total sugar, and HFCS 20% *vs*. both 10% groups for added sugar. No changes were observed in intake of dietary fat (*p* > 0.05).

### 3.3. Body Composition

Changes in body mass and measures of adiposity and body composition are presented in Table 4. While there were no significant changes in body weight for any individual group, a slight increase in body weight was observed when assessing the entire cohort (mean = 2.2 ± 4.9 lbs, *p* < 0.01). While this was statistically significant, during the screening of prospective participants being “weight stable” was defined as there being no change in weight greater than 5 lbs during the past 30 days. Such a change in weight as observed in the present study would therefore still be classified as being “weight-stable” according the criteria used for screening participants. This observed increase in weight represented an increase in percent body fat and fat mass (*p* < 0.01 for both). Importantly changes in these measures were comparable across the 4 groups suggesting group assignment did not influence the degree of weight or fat gain.

**Table 2:** Baseline participant characteristics.

Participants	All	HFCS 10%	HFCS 20%	Sucrose 10%	Sucrose 20%
	(*n* = 65, M = 34, F = 31)	(*n* = 17, M= 9, F= 8)	(*n* = 17, M = 8, F = 9)	(*n* = 18, M = 12, F = 6)	(*n* = 13, M = 5, F = 8)
	Mean ± S.D	Mean ± S.D	Mean ± S.D	Mean ± S.D	Mean ± S.D
Age (years)	39.12 ± 11.76	39.82 ± 11.60	39.33 ± 10.94	41.15 ± 12.24	36.48 ± 12.50
Gender (%)					
Female	52	53	47	67	38
Male	48	47	53	33	62
Race (%)	Caucasian = 68	Caucasian = 71	Caucasian = 68	Caucasian = 72	Caucasian = 69
	African American = 9 Hispanic = 14 Asian = 6 Other = 3	African American = 12 Hispanic = 12 Other = 6	African American = 12 Hispanic = 18 Asian = 12	African American = 11 Hispanic = 6 Asian = 11	African American = 23Other = 8
Height (inches)	66.37 ± 3.77	66.71 ± 4.79	66.30 ± 3.66	66.74 ± 4.05	65.37 ± 3.71
Body Weight (lbs)	179.55 ± 36.12	180.66 ± 40.98	180.31 ± 38.36	178.30	178.87 ± 33.37
BMI	28.58 ± 4.31	27.79 ± 4.30	29.43 ± 3.51	27.96 ± 3.97	29.30 ± 3.88
Waist Circumference (cm)	89.51 ± 11.71	89.16 ± 12.63	91.04 ± 12.75	88.67 ± 11.64	89.18 ± 10.39
Body Fat %	36.31 ± 8.19	36.12 ± 7.74	38.63 ±7.51	32.75 ± 8.91	37.79 ± 7.90
Systolic Blood Pressure (mmHg)	112.46 ± 13.53	111.36 ± 12.81	112.10 ± 14.21	116.00 ± 16.58	110.76 ± 10.81
Diastolic Blood Pressure (mmHg)	73.60 ± 11.55	70.50 ± 15.61	73.48 ± 10.87	75.15 ± 10.99	75.35 ± 7.50
Cholesterol (mg/dL)	183.60 ± 36.61	196.77 ± 39.53	178.24 ± 30.99	175.00 ± 38.25	183.39 ± 34.42
Triglycerides (mg/dL)	128.60 ± 73.61	122.05 ± 57.34	126.48 ± 69.99	121.05 ± 67.09	143.39 ± 95.55
High Density Lipoprotein (mg/dL)	51.13 ± 14.36	53.09 ± 13.40	48.24 ± 15.63	52.70 ± 15.81	50.57 ± 29.64
Low Density Lipoprotein (mg/dL)	106.95 ± 32.19	118.64 ± 37.71	104.67 ± 23.76	98.10 ± 34.68	105.56 ± 29.64

M: male; F: female.

**Table 3:** Changes in macronutrient profile of the diet after ten weeks of sugar sweetened milk consumption as part of a eucaloric diet.

Variable	Time	HFCS 10%	HFCS 20%	Suc 10%	Suc 20%	All
Energy Intake	Baseline	2204 ± 1076	2115 ± 477	2542 ± 1407	2086 ± 964	2260 ± 1048
(kcal)	Week 10	2588 ± 758	2546 ± 836	2970 ± 1786	2328 ± 686	2644 ± 1160 ***
		Interaction *p* = 0.925				
Carbohydrate	Baseline	273.6 ± 125.1	255.9 ± 62.6	323.4 ± 178.2	236.6 ± 102.3	277.2 ± 129.2
(g)	Week 10	341.6 ± 97.5	374.3 ± 132.2	383.0 ± 242.2	348.6 ± 102.0	363.4 ± 158.8 ***
		Interaction *p* = 0.389				
Fat	Baseline	83.5 ± 57.2	82.2 ± 26.5	94.9 ± 58.5	90.8 ± 53.7	87.7 ± 49.8
(g)	Week 10	86.2 ± 38.2	72.2 ± 30.7	99.4 ± 66.9	62.2 ± 23.4	82.3 ± 46.4
		Interaction *p* = 0.074				
Protein	Baseline	89.6 ± 28.2	91.8 ± 22.7	99.7 ± 59.7	85.9 ± 39.2	92.5 ± 40.1
(g)	Week 10	122.0 ± 31.9	109.1 ± 33.8	144.2 ± 69.5	108.0 ± 29.7	122.7 ± 47.6 ***
		Interaction *p* = 0.165				
Total Sugar	Baseline	126.4 ± 65.9	110.6 ± 43.2	154.4 ± 121.0	108.9 ± 69.2	127.5 ± 82.6
(g)	Week 10	199.6 ± 56.1 ***	247.9 ± 99.5 ***	218.8 ± 144.6 **	215.4 ± 83.6 ***	220.5 ± 102.7 ***
		Interaction *p* < 0.05				

Note: ** Different within group than baseline, *p*< 0.01; *** different within group than baseline, *p*< 0.001.

**Table 4:** Changes in body mass, body composition and waist circumference after ten weeks of sugar sweetened milk consumption as part of a eucaloric diet.

Variable	Time	HFCS 10%	HFCS 20%	Suc 10%	Suc 20%	All
Body Mass	Baseline	185.4 ± 44.3	184.5 ± 35.3	179.3 ± 34.4	182.2 ± 39.9	182.8 ± 37.7
(lbs)	Week 10	186.2 ± 43.9	187.9 ± 36.0	181.4 ± 33.9	184.4 ± 41.1	185.0 ± 37.9 **
		Interaction *p* = 0.507				
Waist	Baseline	90.4 ± 13.5	90.9 ± 14.0	88.6 ± 11.2	90.2 ± 11.6	90.0 ± 12.4
(cm)	Week10	90.2 ± 13.2	91.8 ± 14.2	89.5 ± 11.1	90.6 ± 12.1	90.5 ± 12.4
		Interaction *p* = 0.678				
Body Fat	Baseline	35.6 ± 7.3	38.7 ± 7.2	32.6 ± 9.2	37.7 ± 4.8	36.0 ± 7.7
(%)	Week 10	35.8 ± 7.3	38.8 ± 7.0	33.8 ± 9.1	38.1 ± 5.0	36.5 ± 7.5 **
		Interaction *p* = 0.075				
Fat Mass	Baseline	64.2 ±23.5	68.0 ± 15.5	57.3 ± 23.0	66.4 ± 16.2	63.7 ± 20.1
(lbs)	Week 10	64.8 ± 23.0	69.5 ± 15.9	59.7 ± 22.6	68.2 ± 17.7	65.3 ± 20.1 **
		Interaction *p* = 0.532				
Fat Free Mass	Baseline	120.3 ± 27.0	115.3 ± 28.5	121.7 ± 21.7	114.3 ± 26.3	118.1 ± 26.0
(lbs)	Week 10	120.9 ± 29.3	116.9 = 28.1	120.8 ± 22.0	117.3 ± 27.9	118.6 ± 26.3
		Interaction *p* = 0.080				

Note: ** different within group than baseline, *p* < 0.01.

### 3.4. Blood Pressure and Blood Lipids

No changes in systolic (114.12 ± 12.71 *vs*. 113.92 ± 11.52 mmHg, *p* > 0.05) or diastolic blood pressure (73.91 ± 12.01 *vs.* 75.38 ± 9.11 mmHg, *p* > 0.05) were observed, and these parameters were unaffected by the treatment group (interactions *p* = 0.792 and *p* = 0.666, respectively).

Blood lipid data are presented in Table 5. In summary, sweetened milk consumption was seen to result in an overall decrease in HDL (*p* < 0.01) and increase in total cholesterol to HDL ratio (*p* < 0.01), which were unaffected by treatment group (interaction *p* > 0.05). However, changes in total cholesterol, LDL and Apo B were different among the groups—change in HFCS 20% was more positive than that observed in Sucrose 20% However, this was largely driven by an unexpected decrease in this group for these three measures. Triglycerides were unchanged after the 10-weeks (*p* > 0.05).

**Table 5:** Changes in blood lipids after ten weeks of sugar sweetened milk consumption as part of a eucaloric diet.

Variable	Time	HFCS 10%	HFCS 20%	Suc 10%	Suc 20%	All
Total	Baseline	198.3 ± 31.4	178.9 ± 27.5	173.5 ± 35.5	194.2 ± 29.7	185.8 ± 32.3
Cholesterol	Week 10	192.2 ± 30.4	195.4 ± 30.2	174.5 ± 44.5	180.9 ± 24.8 ^†^	186.2 ± 34.1
(mg/dL)		Interaction *p* = 0.019				
Triglycerides	Baseline	123.2 ± 61.7	120.9 ± 61.9	110.3 ± 63.3	166.3 ± 118.5	127.3 ± 76.5
(mg/dL)	Week 10	132.6 ± 39.5	150.9 ± 71.8	118.8 ± 68.1	164.9 ± 85.2	139.8 ± 66.8
		Interaction *p* = 0.585				
HDL	Baseline	53.7 ± 14.8	48.2 ± 14.7	53.7 ± 16.5	50.9 ± 15.2	51.7 ± 15.1
(mg/dL)	Week 10	50.5 ± 14.7	45.4 ± 13.9	52.7 ± 16.6	46.6 ± 11.8	49.0 ± 14.5 **
		Interaction *p* = 0.635				
LDL	Baseline	119.2 ± 29.7	106.5 ± 22.6	97.8 ± 33.1	112.8 ± 26.2	108.9 ± 28.9
(mg/dL)	Week 10	115.1 ± 25.4	119.8 ± 24.9	98.1 ± 36.4	101.3 ± 21.6 ^†^	109.3 ± 28.9
		Interaction *p* = 0.017				
Apolipoprotein	Baseline	100.5 ± 15.1	93.4 ± 19.9	84.7 ± 23.0	100.7 ± 15.2	94.4 ± 19.5
B (mg/dL)	Week 10	100.1 ± 15.5	104.4 ± 20.1 **	88.1 ± 28.1	93.8 ± 20.2 ^†^	96.9 ± 21.4
		Interaction *p* = 0.010				
TC/HDL ratio	Baseline	3.9 ± 0.8	4.0 ± 1.3	3.5 ± 1.1	4.1 ±1.2	3.9 ± 1.1
	Week 10	4.0 ± 0.8	4.6 ± 1.4	3.6 ± 1.5	4.2 ± 1.4	4.1 ± 1.3 **
		Interaction *p* = 0.082				

Note: ** different within group than baseline, *p* < 0.01; ^†^ Change (Week 10-baseline) different than HFCS 20% (*p* < 0.05).

## 4. Discussion

This double blind study examined changes in weight and body composition, blood lipids and blood pressure before and after a 10-week, free living intervention during which low fat (1%) milk was consumed, sweetened with either sucrose or HFCS to deliver 10% or 20% of calories from the sweetener in the context of mixed nutrient meals. Our main findings are: (1) when consumed as part of a eucaloric (weight-stable) diet for a 10-week period, fructose containing sugars do not promote weight gain or an atherogenic lipid profile even when consumed at two to four times the level currently recommended by the AHA [9]; and (2) there are no differences between sucrose and HFCS in these measures at typical levels of sweetener consumption.

Sugars are ubiquitous in the food supply and have been classified as either “naturally occurring” or “added” [9,41,42]. Based on loss-adjusted food availability data from the U.S. Department of Agriculture Economic Research Service [44], the energy contribution from sugars and sweeteners increased by 57 cal/day between 1970 and 2008, while total calories increased 515 cal/day; it should be noted that added fats and cereals/starches increased 231 and 193 cal/day, respectively [44]. The two leading sources of fructose in the American diet are sucrose and HFCS. Worldwide, individuals consume nine times as much fructose in the form of sucrose as is consumed in the form of HFCS [8].

In the current study, baseline sugar consumption for the cohort was slightly above population norms (baseline sugar consumption = 127.5 ± 82.6 g/day). Total sugar consumption increased by approximately 60 grams/day in both the 10% HFCS and 10% sucrose and by over 100 grams per day in the HFCS 20% arm and sucrose 20% arm. As already indicated, individuals followed an overall American Diabetes Association Exchange Diet. They were counseled to substitute the sweetened milk for other carbohydrate exchanges within this diet. When increases in added sugars were assessed it is apparent that individuals in the HFSC and sucrose 10% arm were more able to substitute the sweetened milk for other carbohydrates in their diets when compared with the HFCS and sucrose 20% groups.

While we cannot be certain that the target of 10% or 20% total energy as sucrose or HFCS was attained during the intervention, these increases, coupled with the high level of adherence observed on daily and weekly dietary checklists, suggests that the targets were largely met.

Randomized clinical feeding trials have shown inconsistent results from testing the effects of added sugars on weight gain [27,28,45,46,47,48,49]. Differences in populations studied, study design, study instruments and methods may have contributed to these inconsistencies. Studies utilizing a model of pure fructose versus pure glucose have demonstrated differences in their effects on short term energy regulating hormones such as insulin, leptin and ghrelin [33,34,35]. It has been argued that differences in metabolism between fructose and glucose in the liver could contribute to increased appetite and calories consumed following fructose consumption thereby potentially stimulating weight gain and obesity [33,34,35]. It should be emphasized that neither fructose nor glucose is consumed in isolation to any appreciable degree. Studies which have compared the normally consumed sugars of HFCS and sucrose have not found differences in energy regulating hormones [50,51]. In the current study, no significant changes in body weight, body fat or waist circumference were found in any individual group following a 10-week, free living experiment where either 10% or 20% of calories consumed came from added sugars in the form of sucrose or HFCS.

While the mean weight gain for the entire cohort of 2.2 lbs was technically within the “weight stable” range as defined by this study, it nonetheless bears further scrutiny since it suggests that individuals may have had difficulty incorporating 10% or 20% of calories from added sugars into their normal diets. The average weight gain of 2.2 lbs. over the 10-week study is consistent with the average daily increase of 384 kcals when all four groups were combined. This weight gain is also consistent with results other investigators have reported when adding 25% of calories as either fructose or glucose in free living 10 week studies [33].

Some evidence suggests that increased intake of added sugars may contribute to elevated blood pressure [10,11,12]. These studies include animal trials where rodents were fed extraordinary doses of fructose (greater than 60% of energy), acute ingestion studies where humans were fed high doses of different sugars (25% of energy as fructose or glucose) [33,34,35], and epidemiologic studies such as the Framingham Heart Study [52] and the Nurses Health Trial [53], where consumption of one or more sugar sweetened soft drink per day increased the odds of developing high blood pressure. Results of human studies, however, are inconsistent and very few data are available from randomized, prospective trials at normal population consumed levels of added sugars. A recent meta-analysis of controlled feeding trials found that isocaloric substitution of fructose for other carbohydrates in humans did not adversely affect blood pressure [54].

Lipid abnormalities such as elevation of fasting plasma triglycerides have been demonstrated in diets high in sucrose and fructose (greater than 20% of energy) [55,56]. Fasting triglyceride elevations from sucrose or fructose may be more marked in men than women [18], sedentary or obese individuals [57], and in those with the metabolic syndrome [58]. Recent systematic reviews and meta-analyses, however, have reported that when fructose is substituted isocalorically for other carbohydrates neither fasting [59] or postprandial [60] triglycerides rise. Findings are inconsistent on the effects of added sugars on LDL-cholesterol levels [53,54,55].

In the current study, there was an overall decrease in HDL (*p* < 0.01) and increase in total cholesterol to HDL ratio (*p* < 0.01), however these measures were unaffected by treatment group (interaction *p* > 0.05). Triglycerides rose approximately 10% which was not significant. These changes are all consistent with a diet that replaces dietary fat with carbohydrates, particularly simple sugars. Diets such as the Dietary Approaches to Stop Hypertension (DASH) Diet that replace fat with carbohydrate from fruits, vegetables, whole grains and non-fat and low-fat dairy products that lower HDL cholesterol somewhat but do not increase triglycerides [17,61]. It is also possible that the failure to observe an increase in plasma triglycerides in the overall cohort was somewhat dependent on the slightly elevated baseline sugar consumption, however, as already noted, total sugar consumption rose appropriately in all four groups.

The observed response in triglycerides in HFCS 20% was not a surprising observation. Increased intake of carbohydrates has been shown to promote formation of nascent very-low-density lipoprotein (VLDL) particles by combining glycerol, free fatty acids and Apo B, thus increasing plasma triglycerides Although our study was not designed the explore the hepatic synthesis of VLDL the increased Apo B observed in this group supports our speculation.

It should also be noted that the sugars were delivered in 1% milk and as a result total protein intake also increased. This may have altered the food intake and also hepatic lipid metabolism. Thus, our reported results, related to lipid parameters must be treated with some caution.

In the current study there were no changes in either systolic or diastolic blood pressure (*p* > 0.05) and these parameters were not affected by treatment group. These findings are different from some studies that have suggested that added sugars may raise blood pressure. Moreover, some epidemiologic studies such as the Framingham Heart Study have shown that consumption of ≥1 sugar sweetened carbonated soft drink per day increases the risk of developing high blood pressure [52]. Other studies have not demonstrated elevated blood pressure from increased sugar intake. Thus, the effects of intakes of simple sugars, particularly at average levels on blood pressure remain uncertain.

A strength of the current study is that it is a double blind, randomized, prospective study with a relatively large sample size which explored normal population consumed levels of fructose as delivered through the normally consumed sweeteners, sucrose or HFCS. A further strength of this study is that it examined overweight and obese subjects who are more prone to the metabolic abnormalities assessed in the current investigation. Weaknesses of the current study include that subjects were only followed for 10 weeks and that children, adolescents and individuals over the age of 60 were excluded.

Further studies employing larger numbers of subjects, different population groups (e.g., adolescents and individuals over the age of 60) may be warranted.

## 5. Conclusions

In summary, consumption of added sugars, either from sucrose or HFCS at 10% and 20% of calories consumed (normal population consumption levels) in this 10 week trial, showed no significant changes in any group with respect to weight, adiposity, or abdominal adiposity, and no adverse effects on triglycerides, LDL, or blood pressure in a randomized, controlled trial of free living individuals lasting 10-week. These findings are consistent with other recent studies showing metabolic similarities between HFCS and sucrose [51,62,63,64,65,66,67,68,69].

Although no individual group gained significant weight, the slight weight gain when considering the entire cohort and the mixed response to lipid parameters, such as HDL and total cholesterol, warrant further trials of longer duration and larger number of subjects.

## Availability of Supporting Data

The data set supporting the results of this article is included within the article.
